# 10-Year Risk Estimation for Type 2 Diabetes Mellitus and Coronary Heart Disease in Kuwait: A Cross-Sectional Population-Based Study

**DOI:** 10.1371/journal.pone.0116742

**Published:** 2015-01-28

**Authors:** Abdelmoneim Ismail Awad, Fatemah Mohammad Alsaleh

**Affiliations:** Department of Pharmacy Practice, Faculty of Pharmacy, Kuwait University, Kuwait; University of Tolima, COLOMBIA

## Abstract

**Background:**

Type 2 diabetes mellitus (T2DM), coronary heart disease (CHD) and metabolic syndrome (MetS) are major healthcare problems in Kuwait. The present study was designed to determine the prevalence of MetS, and to estimate the 10-year risk for developing T2DM and CHD among the general population in Kuwait.

**Methods:**

A descriptive, cross-sectional survey was undertaken in 1800 individuals without diabetes or a history of cardiovascular disease (CVD). They were selected from six governorates using two stage convenience sampling. The questionnaire was developed using the Finnish Diabetes Risk Score (FINDRISK), Framingham Risk Score [FRS] and the 2009 Joint Statement criteria for diagnosis of MetS as a framework. Descriptive and multivariate logistic regression analyses were used.

**Results:**

The response rate was 89.4%. More than half (60.8%; 95% CI: 58.4–63.2) of responders were either overweight or obese. One hundred and ninety seven (12.2%) subjects had blood pressure (BP) ≥ 140/90 mm Hg. Almost three-in-ten (28.3%: 26.2–30.6) subjects had fasting plasma glucose (FPG) levels ≥ 5.6 mmol/l, of whom 86.0% and 14.0% had impaired fasting glucose (IFG) and screen detected T2DM, respectively. MetS was present in 512 (31.8%; 29.5–34.2) respondents. Just under one third (n = 481; 29.9%; 27.7–32.2) of participants were at moderate, high, or very high risk of developing T2DM, while 283 (17.6%: 15.8–19.6) were at moderate/high 10-year risk of developing CHD. Approximately one-in-ten (8.5%; 7.2–9.9) subjects were at moderate/high/very high 10-year risk of developing both T2DM/CHD. T2DM risk was higher for females compared to males (p < 0.001); however, the pattern was reversed in terms of the risk of developing CHD or T2DM/CHD. The risk of developing T2DM, CHD, or T2DM/CHD was greater among those aged ≥ 45 years, and those having MetS (p<0.001).

**Conclusions:**

The current findings highlight the need for multifaceted interventions for prevention.

## Introduction

Diabetes is one of the most challenging healthcare problems worldwide, with 382 million individuals aged 20 to 79 years having diabetes in 2013; almost half of them were between 40 and 59 years of age. This prevalence rate is estimated to reach 592 million by 2035. Three of the top 10 countries worldwide with the highest prevalence of diabetes are in the Middle East region: Saudi Arabia (24.0%), Kuwait (23.3%) and Qatar (22.9%) [1]. Kuwait occupies the ninth place among the ten highest ranking countries. The high prevalence of type 2 diabetes mellitus (T2DM) is mainly attributed to dramatic social and culture changes, ageing, urbanization, obesity, decreased physical activity and unhealthy human behaviours [1, 2]. Diabetes poses a great medical and financial burden to many nations worldwide; it reduces life expectancy and quality of life of patients, and imposes a significant burden for society due to increased healthcare costs. In 2013, T2DM-related mortalities were about 5.1 million people aged 20–79 years, representing 8.4% of all global mortalities for individuals in this age group [1]. It has been documented that T2DM is increasing in younger people, especially those aged less than 30 years [3]. Furthermore, T2DM is characterized by a gradual onset and is often asymptomatic for up to 10 years [4], with 20–30% of patients developing complications at the time of diagnosis [5, 6].

There is increasing evidence suggesting that the prevention or delay of T2DM is possible through lifestyle modifications with diet and physical activity, or with pharmacological intervention, especially if implemented before disease manifestation and diagnosis [7, 8]. The earlier identification of high risk subjects is crucial, and a high priority for primary prevention [1, 9]. Thus, several diabetes risk score tools have been described to identify subjects at risk for T2DM [10]. The Finnish Diabetes Risk Score (FINDRISK) is a simple, validated and practical screening tool, which has been successfully used in previous studies to estimate the 10-year risk of T2DM in different populations[10–19]. The wide applicability of FINDRISK is due its focus on general globally prevalent risk factors for T2DM [11].

Cardiovascular diseases (CVDs) are the leading cause of deaths in both developed and developing countries. In 2008, 30% (17.5 million people) of global all-cause mortalities were from CVDs [20]. Of these, 6.2 and 7.3 million were due to stroke and coronary heart disease (CHD), respectively. It is expected to increase to 23.3 million by 2030 [20]. In Kuwait, CHD is the major cause of morbidity and mortality and CVDs are estimated to cause 46.0% of all mortalities [21, 22]. Many risk factors are related to the development of CHD, most importantly are diabetes, obesity, hypertension, hypercholesterolemia, smoking and inactive lifestyle [23, 24]. A significant decrease in the prevalence of CHD can be achieved through the development of effective primary preventive interventions to identify high-risk individuals. Several scoring systems exist to determine the 10-year CHD risk; with the Framingham Risk Score [FRS] being the most widely used and recommended tool to guide the initiation of diagnostic testing and/or primary prevention therapy [25]. It has been shown to be effective in estimating the 10-year risk for developing CHD in different populations [26–31].

MetS is a cluster of cardiovascular risk factors. Patients diagnosed with this syndrome usually have three or more of the following dysmetabolic abnormalities: elevated fasting plasma glucose (FPG), high blood pressure (BP), elevated triglycerides (TG), low high-density lipoprotein cholesterol (HDL-C), and central adiposity that is evident as an increased waist circumference (WC) [32]. It is a high risk condition that can result in serious complications, including T2DM and CVD. In spite of ethnic diversity, MetS doubles the risk of CVD and increases the risk of diabetes by fivefold [32–34]. MetS represents a public health problem necessitating the enhancement of people’s awareness, by the public health authorities, about the importance of healthy lifestyles to reduce obesity and increase physical activity. Furthermore, individuals with MetS need to be identified so that their multiple risk factors and disease incidents can be addressed and decreased [32].

Previous studies reported high prevalence of MetS in Kuwait, ranging from 32.8–40.8% [35–38]. Of these studies, two were conducted in 2001 and 2002, both had evaluated the prevalence in groups of patients attending primary healthcare facilities, which leads to selection bias of the sample [35, 36]. Furthermore, both studies used the 2001 National Cholesterol Education Program's Adult Treatment Panel III (NCEP/ATP III) criteria to identify patients with MetS. The other studies were performed in the years 2006 and 2008–2009, and identified MetS according to the 2006 International Diabetes Federation (IDF) criteria [37, 38]. Since then, various public health efforts have been made to reduce the burden of MetS; therefore, it is important to compute updated prevalence among the general public and determine whether this prevalence has changed. Furthermore, none of the published studies from Kuwait used the 2009 Joint Scientific Statement-Harmonizing the MetS definition.

Yet, little is known about the 10-year risk distribution for T2DM in Kuwait. Only one study was conducted in 2007 to develop a national risk score to predict undiagnosed diabetes and to assess the performance of previously published diabetes risk scores among 560 Kuwaiti patients [39]. It reported a prevalence rate of 4.1% for undetected diabetes and 21.5% for impaired fasting glucose (IFG). The study concluded that the national screening tool identified people at high risk of developing T2DM with better specificity than the Rotterdam, Thai and American Diabetes Association risk scores. A recommendation was made to validate the developed national risk tool in a larger sample size of the Kuwaiti population. To the best of our knowledge, only two studies used FINDRISK in the Middle East region [13, 15], thus our study will be the first in Kuwait and the third in the region.

MetS, CHD and T2DM share common modifiable risk factors. Ideally, identification of individuals with MetS and those who are at risk of developing CHD and T2DM is vital to delay or even prevent the development of T2DM and modify the CHD risk levels. In Kuwait, there are no published data estimating the 10-year risk for developing T2DM using the FINDRISK, and CHD using the FRS among the public. Hence, the present study was designed to explore the recent prevalence of modifiable cardiovascular risk factors, determine the prevalence of MetS, and to estimate the distribution of 10-year risk for developing T2DM and CHD among the general population in Kuwait.

## Materials and Methods

### Study design and population

A descriptive, cross-sectional survey was conducted in Kuwait, a Middle-Eastern country with an area of 17,820 km^2^ and an estimated population of 3,065,850 people; 35.6% of whom are Kuwaitis (2011 estimate) [40]. The study was conducted from March 2012 to May 2013. The study population consisted of Kuwaiti and non-Kuwaiti individuals from six governorates of Kuwait: Al-Ahmadi, Al-Farwaniyah, Al-Jahra, Capital, Hawalli and Mubarak Al-Kabeer. Ethical approval for this study was obtained from the "Human Ethical Committee, Health Sciences Center, Kuwait University".

The sample size was determined using PS power and sample size calculator V.3.05 [41]. A sample of 1080 individuals would be necessary to determine a 10% difference in proportion between two groups; for example, male versus female with 90% power and at 5% significance level. Assuming a response rate of 60%, a sample size of 1800 was selected using two stage convenience sampling. In the first stage, all the six governorates in Kuwait were included in the study. The total number of subjects selected from each governorate was proportional to the population: Al-Farwaniyah, (480), Hawalli (390), Al-Ahmadi (345), Al-Jahra (240), Capital (195), and Mubarak Al-Kabeer (150). In the second stage, the sampling frame was obtained consisting of all suburbs in each of these governorates. The number of subjects selected from each of the suburbs within each governorate was proportional to the population. Subjects in each of the suburbs were approached by pharmacy students and researchers who permanently reside in the respective suburb. They contacted their families, relatives, friends and neighbours to take part in the study. Individuals were given an explanation with regard to the purpose of the study and what was required from them to participate. For those who provided written consent to take part in the study, physical measurements, and venous blood samples for measurement of biochemical data were required to be done at healthcare centres. Participants were assured of confidentiality and were advised of their right to withdraw at any time of the study. Exclusion criteria were age < 20 years and ≥ 80 years, diabetes, CVDs (coronary heart disease, cerebrovascular disease, peripheral vascular disease and heart failure), and chronic kidney disease.

### Study instruments and data collection

The study questionnaire was developed using the FINDRISK and FRS assessment tools, and the 2009 Joint Statement criteria for diagnosis of MetS [12, 25, 32]. The questionnaire consisted of four sections, containing both open-ended and closed-ended questions. The first section included nine items to provide information about the characteristics of the respondents (age, gender, nationality, marital status, educational level, employment, residence, monthly income and personal health). Section two consisted of questions about height, weight and WC measurements. The third section included questions on BP, FPG, total cholesterol (TC), HDL-C and TG measurements. The last section contained ten questions: the first four were about smoking, chronic diseases, and chronic use of drugs, while the remaining six were from the FINDRISK questionnaire relating to daily physical activity ≥ 30 minutes; daily consumption of vegetables, fruits/berries; history of antihypertensive drug treatment, high blood glucose levels and family history of T2DM. The questionnaire was translated into Arabic and subjected to a process of forward and backward translation. The accuracy and meaning of the translated versions were checked prior to data collection, the questionnaire was pretested for content, design, readability, and comprehension in 10 subjects.

Each participant was asked to complete the first and fourth sections of the questionnaire, and to obtain physical measurements of BP (average of 2 measures in the sitting position), weight, height and WC; and the results of a fasting biochemical data for plasma glucose, TC, HDL-C and TG. They were informed and consented to fast for 12 hours prior to blood testing. These measurements were done at the healthcare centers in the participants’ residential area, and were returned to the data collectors to complete the second and third sections of the questionnaire.

### Identification of individuals with MetS

The study subjects were assessed according to the 2009 Joint Scientific Statement-Harmonizing the MetS definition [32]. Three out of the following five criteria would qualify a subject for a diagnosis of MetS: raised TG level (≥ 1.7 mmol/l); reduced HDL-C (< 1.0 mmol/l in males and < 1.3 mmol/l in females) or specific treatment for these lipid abnormalities; raised systolic ≥ 130 and/or diastolic BP ≥ 85 mm Hg or treatment of previously diagnosed hypertension; raised FPG ≥ 5.6 mmol/l or previously diagnosed T2DM. Europid cut-points were used to identify central obesity (WC: male ≥ 94 cm, female ≥ 80 cm) according to the IDF recommendations due to lack of specific cut-points for the Middle-East population [32].

### Identification of overweight and obese individuals

Body mass index (BMI) was calculated by dividing the weight in kilograms by height in meters squared. The participants were classified as overweight (BMI 25–29.9 kg/m2) or obese (BMI ≥ 30 kg/m^2^) according to the World Health Organization (WHO) definition [42].

### Identification of individuals with abnormal plasma glucose levels

Glucose tolerance was classified according to the American Diabetes Association (ADA) criteria for diagnosis and classification of diabetes [43]. Subjects who were not known to have diabetes and who had FPG ≥ 7.0mmol/l were classified as having screen-detected T2DM. Participants who had FPG between 5.6 to 6.9 mmol/l were classified as having IFG.

### Identification of individuals with abnormal BP

The study participants were categorized as having optimal/normal BP (< 130/85 mm Hg), high normal BP (130–139/85–89mm Hg) and hypertension (≥ 140/90 mm Hg), according to the 2013 European Society of Hypertension /European Society of Cardiology guidelines for the management of arterial hypertension [44].

### Identification of individuals with abnormal total cholesterol, HDL-C and TG

The subjects levels of total cholesterol, HDL-C and TG were categorized as follows: (1) optimal TC (< 4.14 mmol/l), desirable (4.14–5.16 mmol/l) and high (≥ 5.17 mmol/L); (2) low HDL-C for males (< 1.03 mmol/l) and for females (< 1.3 mmol/l); and (3) normal TG (< 1.7 mmol/l), borderline-high (1.7 mmol/l-2.25 mmol/l) and high (≥ 2.26 mmol/l) [25, 32].

### Estimation of the 10-year risk for T2DM using FINDRISK screening tool

FINDRISK is composed of 8 items: age, BMI (kg/m^2^), WC (cm), physical activity, diet, antihypertensive drug use, history of high blood glucose and family history of diabetes. The answer for each question is weighted, according to the increased risk, with a maximum score of 26 points. The risk of developing T2DM within 10 years is categorized according to the risk scores: low (< 7 points), slightly elevated (7–11 points), moderate (12–14 points), high (15–20 points) and very high (> 20 points).

### Estimation of the 10-year risk for CHD using FRS screening tool

FRS is a gender-specific tool that is used to estimate the 10-year risk for hard CHD (myocardial infarction + CHD death) [25], which is a modified version of the risk prediction algorithm from the Framingham Heart study [23]. It includes the patient’s age, TC level, HDL-C level, smoking status, and systolic BP to estimate an individual’s 10-year risk for developing CHD. Three categories of risk are defined: low risk (< 10%), moderate (10%-20%), and high (> 20%) that is categorized as CHD risk equivalent.

The study participants who were found to be with abnormal physical and /or biochemical laboratory parameters, MetS, and moderate/high/very high 10-year risk of developing T2DM or CHD or T2DM/CHD were informed and provided with lifestyle counselling. They were also referred to their general practitioners.

### Statistical analysis

Data were entered into the Statistical Package for Social Sciences (SPSS, version 21, SPSS, Chicago, IL, U.S.A.). The results were reported as percentages (95% confidence intervals; CI), means (standard deviations; SD) and medians (interquartile ranges). The general characteristics and the variables of FINDRISK and FRS tools for responders were compared by gender using Mann-Whitney test for continuous variables and Chi-square test for categorical variables, using p< 0.05 to indicate statistical significance. As the main outcome measure was a binary variable describing the presence of MetS (yes, no), and the 10-year risk of developing T2DM or CHD or T2DM/CHD that was categorized into two levels of low risk and moderate/high/very high risk, logistic regression models were performed using SPSS to fit the best model for the predictor-independent variables. Only the results of multivariate logistic analysis are reported showing odds ratio (OR) and 95% CI. The predictor variables were categorized as follows: (1) gender: males and females; (2) age: young adults [20–44 years], middle age groups [45–54 years] and [55–64 years], and old age [*≥* 65 years]; (3) nationality: Kuwaiti and non-Kuwaiti; (4) level of education (a) low-intermediate [0–12 years] for those who completed secondary school or less and (b) high [>12 years] for those who had diploma, or bachelor degree or postgraduate degree; (5) residence: six governorates of Kuwait (6) monthly income: low [< 500 Kuwaiti Dinars (KD)], middle [500–1000 KD] and high [> 1000 KD]. Due to the convenience sample that was used in this study the distribution of gender and education level were found to be markedly different from that of the population census data. Further analyses using sample weighted logistic regression method were used to adjust for this discrepancy. Weights were computed based on the latest national census data.

## Results

### Study population

A total of 1800 subjects were invited to participate in the study, 1610 agreed to participate, giving a response rate of 89.4%. Reasons for refusals included: no time to visit the healthcare centers to obtain the physical and biochemical laboratory parameters; unwillingness to know their risk of developing T2DM and CHD; regular disease checking; and excellent health. The overall mean (SD) age of respondents was 37.7 (12.4) years. Nine hundred and eighty two (61.0%) were females. Almost one-fifth (n = 314; 19.5%) of participants had chronic diseases, including hypertension (n = 187; 11.6%; 95% CI: 10.1–13.3) and others such as asthma, chronic obstructive pulmonary disease, epilepsy, gout, hypothyroidism, migraine, osteoarthritis and rheumatoid arthritis (n = 127; 7.9%; 95% CI: 6.6–9.3). About seven-in-ten (n = 1117; 69.3%; 95% CI: 67.1–71.6) subjects reported excellent and very good general health. Characteristics of the study subjects are presented in Table 1.

**Table 1:** General characteristics of the study subjects.

Characteristics	n (%, 95% CI)^#^	Gender^*^		Nationality^^^	
		Male	Female	Kuwaiti	Non-Kuwaiti
		n(%)	n(%)	n(%)	n(%)
	n = 1610	n = 628	n = 982	n = 1107	n = 503
**Martial state**					
Single	451 (28.0, 25.8, 30.3)	149 (23.7)	302 (30.8)	343 (31.0)	108 (21.5)
Married	1076 (66.8, 64.5–69.1)	458 (72.9)	618 (62.9)	695 (62.8)	381 (75.7)
Divorced	59 (3.7, 2.8–4.7)	16 (2.6)	43 (4.4)	48 (4.3)	11 (2.2)
Widowed	24 (1.5, 1.0–2.3)	5 (0.8)	19 (1.9)	21 (1.9)	3 (0.6)
**Age (Yrs)**					
20–44	1108 (68.8, 66.5–71.0)	402 (64.0)	706 (71.9)	782 (70.6)	326 (64.8)
45–54	338 (21.0, 19.0–23.1)	134 (21.3)	204 (20.8)	223 (20.1)	115 (22.9)
55–64	132 (8.2, 6.9–9.7)	75 (11.9)	57 (5.8)	83 (7.5)	49 (9.7)
≥ 65	32 (2.0, 1.4–2.8)	17 (2.8)	15 (1.5)	19 (1.7)	13 (2.6)
**Educational level**					
Low-intermediate education	309 (19.2, 17.3–21.2)	119 (18.9)	190 (19.3)	172 (15.5)	137 (27.2)
High education	1301 (80.8, 78.8–82.6)	509 (81.1)	792 (80.7)	935 (84.5)	366 (72.8)
**Residence (Governorates)**					
Capital	187 (11.6, 10.1–13.3)	62 (9.9)	125 (12.7)	155 (14.0)	32 (6.4)
Hawalli	348 (21.6, 19.6–23.7)	131 (20.9)	217 (22.1)	216 (19.5)	132 (26.2)
Al-Farwaniyah	438 (27.2, 25.1–29.5)	177 (28.2)	261 (26.6)	306 (27.6)	132 (26.2)
Al-Ahmadi	317 (19.7,17.8–21.7)	134 (21.3)	183 (18.6)	206 (18.6)	111 (22.1)
Al-Jahra	200 (12.4,10.9–14.2)	75 (11.9)	125 (12.8)	116 (10.5)	84 (16.7)
Mubarak Al-Kabeer	120 (7.5, 6.2–8.9)	49 (7.8)	71 (7.2)	108 (9.8)	12 (2.4)
**Monthly income (KD)**					
< 500	440 (27.3, 25.2–29.5)	143 (22.7)	297 (30.2)	203 (18.3)	237 (47.1)
500–1000	586 (36.4, 34.1–38.8)	165 (26.3)	421 (42.9)	410 (37.0)	176 (35.0)
> 1000	584 (36.3, 33.9–38.6)	320 (51.0)	264 (26.9)	494 (44.6)	90 (17.9)
**Personal health**					
Excellent	532 (33.0, 30.8–35.4)	218 (34.7)	314 (32.0)	423 (38.2)	109 (21.7)
Very good	585 (36.3, 34.0–38.7)	211 (33.6)	374 (38.1)	413 (37.3)	172 (34.2)
Good	443 (27.5, 25.4–29.7)	181 (28.8)	262 (26.7)	248 (22.4)	195 (38.8)
Fair	50 (3.1, 2.3–4.1)	18 (2.9)	32 (3.2)	23 (2.1)	27 (5.4)
**Chronic disease**					
Yes	314 (19.5, 17.6–21.5)	119 (18.9)	195 (19.9)	223 (20.1)	91 (18.1)
No	1296 (80.5, 78.5–82.3)	509 (81.1)	787 (80.1)	884 (79.9)	412 (81.9)
**Smoking**					
Yes	256 (15.9, 14.2–17.8)	229 (36.5)	27 (2.7)	173 (15.6)	83 (16.5)
No	1354 (84.1, 82.2–85.8)	399 (63.5)	955 (97.3)	934 (84.4)	420 (83.5)

n: Number of participants;

#: Overall percentage;

*: Percentage within gender;

^: Percentage within nationality;

CI: Confidence interval; Yrs: years; KD: Kuwaiti Dinars

One hundred and ninety seven (12.2%) subjects had BP ≥ 140/90 mm Hg, of whom 62 (31.5%) had hypertension and were taking antihypertensive medications, and 135 (68.5%) with undetected hypertension. The overall prevalence of hypertension among participants was 20.0% (95% CI: 18.1–22.1%), of these 187 (11.6%) reported a previous diagnosis of hypertension and 135 (8.4%; 95% CI: 7.1–9.9) were classified as having screen-detected hypertension. One quarter (n = 405; 25.2%) and almost three-quarters (n = 1188; 73.8%) of participants had optimal TC (< 4.14 mmol/l) and TG (< 1.7 mmol/l), respectively. HDL-C levels were low (< 1.3 for females; < 1.03 for males) in 49.1% of the females and 37.4% of the males. Just less than one third (n = 456; 28.3%) subjects had FPG levels ≥ 5.6 mmol/L. Three hundred and ninety two (24.4%; 95% CI: 22.3–26.5) and 64 (4.0%; 95% CI: 3.1–5.1) subjects were classified as having IFG (5.6–6.9 mmol/l) and screen-detected T2DM (≥ 7.0 mmol/l), respectively. Forty two (9.2%) of them were taking drugs that can elevate blood glucose levels, including beta-blockers, hydrochlorothiazide, levothyroxine and prednisolone. A history of high episodes of blood glucose levels was reported by 82 (18.0%) of whom had FPG ≥ 5.6 mmol/l, five of them were using levothyroxine.

Table 2 shows the physical and biochemical laboratory data of the study participants. About 60% (n = 979; 95% CI: 58.4–63.2) of responders were classified as either overweight or obese (BMI ≥ 25 kg/m^2^), of whom 348 (35.5%) were obese (BMI ≥ 30 kg/m^2^). WC were high (≥ 80 cm for females; ≥ 94 cm for males) in 65.1% (95% CI: 62.1–68.1) of the females and 41.4 (95% CI: 37.5–45.4) of the males. Daily physical activity of ≥ 30 minutes was reported by 979 participants (60.8%). Irregular vegetables and fruit consumption was indicated by more than half of the respondents (n = 848; 52.7%). About 70% (n = 1158) individuals had a family history of diabetes. Table 3 presents the distribution of FINDRISK variables of the study’s participants.

**Table 2:** Physical and biochemical laboratory data of the study subjects.

Physical and laboratory characteristics	n (%, 95% CI)^#^	Gender^*^		Nationality^^^	
		Male	Female	Kuwaiti	Non-Kuwaiti
		n(%)	n(%)	n(%)	n(%)
	n = 1610	n = 628	n = 982	n = 1107	n = 503
**Systolic BP (mm Hg)**					
< 130	1115 (69.3, 66.9–71.5)	376 (59.9)	739 (75.2)	789 (71.3)	326 (64.8)
130–139	365 (22.7, 20.7–24.8)	170 (27.1)	195 (19.9)	252 (22.7)	113 (22.5)
≥ 140	130 (8.1, 6.8–9.5)	82 (13.0)	48 (4.9)	66 (6.0)	64 (12.7)
**Diastolic BP (mm Hg)**					
< 85	1315 (81.7, 79.7–83.5)	469 (74.7)	846 (86.2)	929 (83.9)	386 (76.8)
85–89	160 (9.9, 8.5–11.5)	81 (12.9)	79 (8.0)	111 (10.0)	49 (9.7)
≥ 90	135 (8.4, 7.1–9.9)	78 (12.4)	57 (5.8)	67 (6.1)	68 (13.5)
**BP (mm Hg)**					
< 130/85	1043 (64.8, 62.4–67.1)	339 (54.0)	704 (71.7)	741 (66.9)	302 (60.0)
≥ 130–139/85–89	370 (23.0, 21.0–25.1)	171 (27.2)	199 (20.3)	267 (24.2)	103 (20.5)
≥ 140/90	197 (12.2, 10.7–13.9)	118 (18.8)	79 (8.0)	99 (8.9)	98 (19.5)
**TC (mmol/L)**					
< 4.14	405 (25.2, 23.1–27.3)	154 (24.5)	251 (25.6)	287 (26.0)	118 (23.4)
4.14–5.16	649 (40.3, 37.9–42.7)	254 (40.5)	395 (40.2)	451 (40.7)	198 (39.4)
≥ 5.17	556 (34.5, 32.2–36.9)	220 (35.0)	336 (34.2)	369 (33.3)	187 (37.2)
**HDL-C**					
**Men**					
< 1.03		235 (37.4)		140 (36.8)	95 (38.3)
≥ 1.03		393 (62.6)		240 (63.2)	153 (61.7)
**Women**					
< 1.3			482 (49.1)	340 (46.8)	142 (55.7)
≥ 1.3			500 (50.9)	387 (53.2)	113 (44.3)
**TG (mmol/L)**					
< 1.7	1188 (73.8, 71.6–75.9)	424 (67.5)	764 (77.8)	837 (75.6)	351 (69.8)
1.7–2.25	212 (13.2, 11.6–14.9)	101 (16.1)	111 (11.3)	138 (12.5)	74 (14.7)
≥ 2.26	210 (13.0, 11.5–14.8)	103 (16.4)	107 (10.9)	132 (11.9)	78 (15.5)
**FPG (mmo/L)**					
< 5.6	1154 (71.7, 69.4–73.8)	428 (68.2)	726 (73.9)	814 (73.5)	340 (67.6)
≥ 5.6	456 (28.3, 26.2–30.6)	200 (31.8)	256 (26.1)	293 (26.5)	163 (32.4)

n: Number of participants;

#: Overall percentage;

*: Percentage within gender;

^: Percentage within nationality;

CI: Confidence interval; Kg: Kilogram; m: Meter; BP: Blood pressure; mm Hg: Millimeter mercury; TC: Total cholesterol; HDL-C: High density lipoprotein cholesterol; TG: Triglycerides; FPG: Fasting plasma glucose; mmol/L: Millimoles per liter.

**Table 3:** Distribution of FINDRISK variables of the study subjects.

Variable	n (%, 95% CI)^#^	Gender^*^		Nationality^^^	
		Male	Female	Kuwaiti	Non-Kuwaiti
		n(%)	n(%)	n(%)	n(%)
	n = 1610	n = 628	n = 982	n = 1107	n = 503
**BMI (kg/m^2^)**					
< 25	631 (39.2, 36.8–41.6)	209 (33.3)	422 (43.0)	449 (40.5)	182 (36.2)
25–30	631 (39.2, 36.8–41.6)	296 (47.1)	335 (34.1)	419 (37.9)	212 (42.1)
>30	348 (21.6, 19.6–23.7)	123 (19.6)	225 (22.9)	239 (21.6)	109 (21.7)
**Waist Circumference (cm)**					
**Men**					
< 94		368 (58.6)		206 (54.5)	162 (64.8)
94–102		137 (21.8)		89 (23.5)	48 (19.2)
>102		123 (19.6)		83 (22.0)	40 (16.0)
**Women**					
<80			342 (34.9)	272 (37.3)	70 (27.7)
80–88			239 (24.3)	169 (23.2)	70 (27.7)
>88			401 (40.8)	288 (39.5)	113 (44.6)
**Daily physical activity ≥ 30 minutes**					
Yes	979 (60.8, 58.4–63.2)	416 (66.2)	563 (57.3)	665 (60.1)	314 (62.4)
No	631 (39.2, 36.8–41.6)	212 (33.8)	419 (42.7)	442 (39.9)	189 (37.6)
**Daily vegetables, fruit or berries consumption**					
Yes	762 (47.3, 44.9–49.8)	289 (46.0)	473 (48.2)	523 (47.2)	239 (47.5)
No	848 (52.7, 50.2–55.1)	339 (54.0)	509 (51.8)	584 (52.8)	264 (52.5)
**Antihypertensive use**					
Yes	187 (11.6, 10.1–13.3)	84 (13.4)	103 (10.5)	118 (10.7)	69 (13.7)
No	1423 (88.4, 86.7–89.9)	544 (86.6)	879 (89.5)	989 (89.3)	434 (86.3)
**History of high blood glucose**					
Yes	146 (9.1, 7.7–10.6)	47 (7.5)	99 (10.1)	91 (8.2)	55 (10.9)
No	1464 (90.9, 89.4–92.3)	581 (92.5)	883 (89.9)	1016 (91.8)	448 (89.1)
**Family history of diabetes**					
Yes	1158 (71.9, 69.7–74.1)	427 (68.0)	731 (74.4)	851 (76.9)	307 (61.0)
No	452 (28.1, 25.9–30.4)	201 (32.0)	251 (25.6)	256 (23.1)	196 (39.0)

FINDRISK: The Finnish Diabetes Risk Score; n: Number of participants;

#: Overall percentage;

*: Percentage within gender;

^: Percentage within nationality;

CI: Confidence interval; BMI: Body mass index; Kg/m^2^: Kilogram per square meter; cm: Centimeter.

Table 4 shows the characteristics of the study’s population by gender. The median (Interquartile range; IQR) of age, height, weight, WC, BP, FPG, TG and BMI was significantly lower among females compared to males (p < 0.05). Compared to males, females had significantly higher HDL-C (p < 0.05). However, the prevalence rates of smoking and daily physical activity ≥ 30 minutes were significantly higher among males (p < 0.05). Family history of diabetes was significantly common among females compared to males (p < 0.05). There was no significant difference in the distribution of other variables.

**Table 4:** General characteristics and variables of FINDRISK and FRS of study subjects by gender (n = 1610).

Characteristic	Male (n = 628)		Female (n = 982)		p-value
	Mean (SD) or Frequency (%)	Median (IQR)	Mean (SD) or Frequency (%)	Median (IQR)	
**Age (Yrs)**	39.8 (12.7)	39.0 (29.0–49.0)	36.4 (12.1)	35.0 (25.0–45.0)	<0.001
**Height (m)**	1.73 (0.08)	1.73 (1.68–1.78)	1.61 (0.07)	1.60 (1.56–1.65)	<0.001
**Weight (kg)**	81.6 (16.0)	80.0 (70.0–90.0)	69.1 (15.1)	67.0 (59.0–78.0)	<0.001
**Waist Circumference (cm)**	92.4 (14.9)	91.4 (81.3–100.0)	86.4 (16.0)	84.0 (75.0–96.0)	<0.001
**Systolic BP (mm Hg)**	125 (12)	125 (120–130)	121 (12)1	120 (112–129)	<0.001
**Diastolic BP (mm Hg)**	80 (7)	80 (78–85)	77 (8)	76 (66–77)	<0.001
**(FPG) (mmo/L)**	5.3 (1.0)	5.3 (4.8–5.7)	5.2 (0.9)	5.1 (4.7–5.6)	0.002
**TC (mmol/L)**	4.9 (1.0)	4.8 (4.1–5.5)	4.9 (1.0)	4.8 (4.1–5.5)	0.687
**HDL-C (mmol/L)**	1.17 (0.31)	1.10 (0.97–1.30)	1.30 (0.34)	1.30 (1.07–1.52)	<0.001
**TG (mmol/L)**	1.5 (0.90)	1.3 (0.9–1.9)	1.2 (0.7)	1.0 (0.7–1.6)	<0.001
**BMI (kg/m^2^)**	27.1 (4.6)	26.6 (24.1–29.3)	26.7 (5.7)	25.8 (22.8–29.7)	0.003
**Educational level**					0.843
Low-intermediate education	119 (18.9)		190 (19.3)		
High education	509 (81.1%)		792 (80.7)		
**Chronic disease**					0.654
Yes	119 (18.9)		195 (19.9)		
No	509 (81.1)		787 (80.1)		
**Smoking**					<0.001
Yes	229 (36.5)		27 (2.7)		
No	399 (63.5)		955 (97.3)		
**Daily physical activity ≥ 30 minutes**					<0.001
Yes	416 (66.2)		563 (57.3)		
No	212 (33.8)		419 (42.7)		
**Daily vegetables, fruit or berries consumption**					0.400
Yes	289 (46.0)		473 (48.2)		
No	339 (54.0)		509 (51.8)		
**Antihypertensive use**					0.078
Yes	84 (13.4)		103 (10.5)		
No	544 (86.6)		879 (89.5)		
**History of high blood glucose**					0.077
Yes	47 (7.5)		99 (10.1)		
No	581 (92.5)		883 (89.9)		
**Family history of diabetes**					0.005
Yes	427 (68.0)		731 (74.4)		
No	201 (32.0)		251 (25.6)		

FINDRISK: The Finnish Diabetes Risk Score; FRS: The Framingham Risk Score; n: Number of participants; CI: Confidence interval; SD: Standard deviation;

IQR: Interquartile range; Yrs: Years; m: Meter; Kg: Kilogram; cm: Centimeter; BP: Blood pressure; mm Hg: Millimeter mercury; FPG: Fasting plasma glucose;

mmol/L: Millimoles per liter; TC: Total cholesterol; HDL-C: High density lipoprotein cholesterol; TG: Triglycerides; BMI: Body mass index; Kg/m^2^: Kilogram per square meter.

MetS was present in about 32% (n = 512; 95% CI: 29.5–34.2) of the participants. Table 5 presents the adjusted OR and 95% CI that quantify the association between subjects who were found to have MetS and their characteristics. MetS was found to be significantly lower among the youngest age group (20–44 years), BMI of < 25 kg/m^2^ and those who reported daily physical activity ≥ 30 minutes (p < 0.001). Weighted logistic regression estimates did not show remarkable differences to that of the unweighted estimates.

**Table 5:** Association between participants who were found to have MetS and their characteristics (n = 512).

Characteristics	OR (95% CI)	p-value	Sample Weighted OR (95% CI)	p-value
**Gender**		0.494		0.754
Male	Reference		Reference	
Female	0.92 (0.71–1.18)		0.93 (0.60–1.45)	
**Age (Yrs)**		<0.001		
20–44	Reference		Reference	0.005
45–54	2.54 (1.92–3.38)		2.34 (1.41–3.86)	
55–64	3.23 (2.14–4.87)		2.46 (1.15–5.28)	
≥ 65	3.16 (1.44–6.95)		2.29 (0.80–6.54)	
**Nationality**		0.098		0.512
Kuwaiti	0.78 (0.58–1.05)		0.85 (0.52–1.39)	
Non-Kuwaiti	Reference		Reference	
**Educational level**		0.867		0.878
Low-intermediate education	1.03 (0.75–1.41)		0.97 (0.65–1.45)	
High education	Reference		Reference	
**Residence (Governorates)**		0.068		0.154
Capital	Reference		Reference	
Hawalli	0.92 (0.59–1.43)		1.18 (0.54–2.54)	
Al-Farwaniyah	1.50 (0.99–2.27)		1.43 (0.69–2.97)	
Al-Ahmadi	1.08 (0.69–1.69)		0.72 (0.33–1.55)	
Al-Jahra	0.74 (0.45–1.22)		0.64 (0.27–1.51)	
Mubarak Al-Kabeer	0.83 (0.46–1.48)		0.70 (0.22–2.26)	
**Monthly income (KD)**		0.610		0.332
< 500	Reference		Reference	
500–1000	0.99 (0.71–1.36)		1.15 (0.64–2.08)	
> 1000	1.10 (0.77–1.57)		0.72 (0.37–1.39)	
**Daily physical activity ≥ 30 minutes**		<0.001		0.022
Yes	Reference		Reference	
No	1.74 (1.37–2.20)		1.67 (1.08–2.59)	
**Daily vegetables, fruit or berries consumption**		0.722		0.817
Yes	Reference		Reference	
No	0.96 (0.76–1.21)		0.95 (0.61–1.48)	
**BMI (kg/m2)**		<0.001		<0.001
**< 25**	Reference		Reference	
**25–30**	2.28 (1.71–3.04)		1.94 (1.15–3.27)	
**>30**	6.77 (4.92–9.33)		4.52 (2.56–8.01)	

MetS: Metabolic syndrome: n: Number of participants; OR: Odds ratio; CI: Confidence interval; KD: Kuwaiti Dinars; Kg/m^2^: Kilogram per square meter.

### Estimation of the 10-year risk of T2DM and/or CHD

The overall median (IQR) FINDRISK risk-score was 9.0 (5.0–12.0) and it was significantly higher among females as compared to males (p < 0.001). Table 6 provides the distribution of FINDRISK risk levels among the study population and probabilities for each risk level of developing T2DM. Almost 30% (n = 481; 95% CI: 27.7–32.2) of participants were found to be at moderate/high/very high risk of developing T2DM within the next 10 years. The 10-year risk of developing CHD in the study subjects per FRS was as follows: low (n = 1327; 82.4%; 95% CI: 80.5–84.2), moderate (n = 250; 15.5%; 95% CI: 13.8–17.4), high (n = 33; 2.1%; 95% CI: 1.4–2.9). One hundred and thirty six (8.45%; 95% CI: 7.2–9.9) were found to be at moderate/high/very high risk of developing both T2DM/CHD within the next 10 years.

**Table 6:** Estimated risk of developing T2DM within 10-years in the study population per FINDRISK (n = 1610).

Individual risk Score	Estimated probability of developing diabetes	Frequency (%, 95% CI)
Low risk (< 7)	1 in 100	566 (35.2, 32.8–37.6)
Slightly elevated risk (7–11)	1 in 25	563 (34.9, 32.7–37.4)
Moderate risk (12–14)	1in 6	269 (16.7, 14.9–18.6)
High risk (15–20)	1 in 3	195 (12.1, 10.6–13.8)
Very high risk (> 20)	1 in 2	17 (1.1, 0.6–1.7)

T2DM: Type 2 diabetes mellitus; FINDRISK: The Finnish Diabetes Risk Score; n: Number of participants; CI: Confidence interval

Table 7 provides the adjusted OR and 95% CI that quantify the association between participants who were found to be at moderate/high/very high risk of developing T2DM, CHD, or both and their characteristics.. The risk of developing T2DM was higher among females compared to males (p < 0.001); however, the pattern was reversed in terms of the risk of developing CHD or both T2DM/CHD. The risk of developing T2DM, CHD, or both was least common among the youngest age group (20–44 years) compared to those aged ≥ 45 years (p < 0.001), and the highest among the elderly aged group (≥ 65 years). Subjects with low-intermediate level of education were found to have a greater risk of developing T2DM compared to those with a high level of education (OR: 1.43; 1.02–2.00); however, there were no significant differences between their risk of developing CHD or both T2DM /CHD (p > 0.05). Low income group was found to have the lowest risk of developing T2DM compared to middle and high income earners (p = 0.001); however, subjects’ income was not significantly associated with the risk of developing CHD or both T2DM/CHD (p > 0.05). Respondents with MetS had a greater risk of developing T2DM, CHD or both (p < 0.001). Table 8 presents the point and interval estimate as well the p values obtained using the sample weighted logistic regression. It should be noted that the associations remain similar to the unweighted analysis except, that the statistically significant difference in the gender for the risk of developing both T2DM/CHD had attenuated compared to the unweighted analysis.

**Table 7:** Association between participants at moderate/high/very high risk to develop T2DM and/or CHD, and their characteristics.

	Moderate/high/very high risk to develop T2DM (n = 481)		Moderate/high to develop CHD (n = 283)		Moderate/high/very high risk to develop both T2DM/CHD (n = 136)	
Characteristics	OR (95% CI)	p-value	OR (95% CI)	p-value	OR (95% CI)	p-value
**Gender**		<0.001		<0.001		0.039
Male	Reference		Reference		Reference	
Female	2.08 (1.58–2.75)		0.22 (0.16–0.32)		0.62 (0.. 39–0.98)	
**Age (Yrs)**		<0.001		<0.001		<0.001
20–44	Reference		Reference		Reference	
45–54	2.75 (2.06–3.69)		10.63 (7.14–15.82)		12.76 (6.76–24.09)	
55–64	3.97 (2.59–6.08)		38.78 (23.27–64.62)		32.14 (16.08–64.23)	
≥ 65	10.16 (4.10–25.14)		42.04 (17.17–94.13)		64.03 (23.41–175.17)	
**Nationality**		0.090		0.245		0.177
Kuwaiti	1.30 (0.96–1.75)		1.26 (0.85–1.87)		1.42 (0.85–2.35)	
Non-Kuwaiti	Reference		Reference		Reference	
**Educational level**		0.038		0.811		0.459
Low-intermediate Education	1.43 (1.02–2.00)		1.05 (0.69–1.1)		1.22 (0.72–2.06)	
High education	Reference		Reference		Reference	
**Residence (Governorates)**		0.419		0.256		0. 766
Capital	Reference		Reference		Reference	
Hawalli	1.50 (0.94–2.39)		0.80 (0.52–1.24)		0.62 (0.30–1.29)	
Al-Farwaniyah	1.16 (0.74–1.83)		1.16 (0.69–1.96)		0.60 (0.29–1.25)	
Al-Ahmadi	1.37 (0.85–2.20)		0.62 (0.32–1.23)		0.75 (0.35–1.59)	
Al-Jahra	1.32 (0.78–2.24)		0.49 (0.19–1.25)		0.66 (0.29–1.54)	
Mubarak Al-Kabeer	1.64 (0.91–2.94)		0.69 (0.36–1.32)		0.87 (0.32–2.38)	
**Monthly income (KD)**		0.001		0.603		0.118
< 500	Reference		Reference		Reference	
500–1000	1.67 (1.18–2.37)		0.79 (0.49–1.27)		0.82 (0.43–1.57)	
> 1000	2.11 (1.45–3.08)		0.81 (0.. 49–1.34)		1.45 (0.75–2.82)	
**MetS**		<0.001		<0.001		<0.001
Yes	5.12 (3.96–6.61)		2.81 (2.01–3.92)		4.82 (3.08–7.53)	
No	Reference		Reference		Reference	

T2DM: Type 2 diabetes mellitus; CHD: Coronary heart disease; n: Number of participants; OR: Odds ratio; CI: Confidence interval; Yrs: Years; KD: Kuwaiti Dinars;

MetS: Metabolic syndrome.

**Table 8:** Association quantified by sample weighted regression between participants at moderate/high/very high risk to develop T2DM and/or CHD, and their characteristics.

	Moderate/high/very high risk to develop T2DM (n = 481)		Moderate/high to develop CHD (n = 283)		Moderate/high/very high risk to develop both T2DM/CHD (n = 136)	
Characteristics	OR (95% CI)	p-value	OR (95% CI)	p-value	OR (95% CI)	p-value
**Gender**		0.001		<0.001		0.061
Male	Reference		Reference		Reference	
Female	2.27 (1.39–3.72)		0.18 (0.10–0.34)		0.48 (0.22–1.04)	
**Age (Yrs)**		<0.001		<0.001		<0.001
20–44	Reference		Reference		Reference	
45–54	2.65 (1.56–4.50)		6.42 (3.11–13.22)		12.50 (3.37–46.40)	
55–64	3.57 (1.80–7.11)		29.40 (11.73–73.72)		35.44 (9.22–136.18)	
≥ 65	14.32 (3.81–53.80)		30.57 (12.10–77.27)		81.40 (16.72–396.37)	
**Nationality**		0.208		0.210		0.679
Kuwaiti	1.40 (0.83–2.36)		0.67 (0.35–1.26)		0.82 (0.32–2.12)	
Non-Kuwaiti	Reference		Reference		Reference	
**Educational level**		0.040		0.084		0.175
Low-intermediate Education	1.53 (1.02–2.30)		1.57 (0.94–2.60)		1.62 (0.81–3.26)	
High education	Reference		Reference		Reference	
**Residence (Governorates)**		0.066		0.753		0. 141
Capital	Reference		Reference		Reference	
Hawalli	2.10 (0.93–4.79)		0.59 (0.15–2.33)		0.92 (0.27–3.13)	
Al-Farwaniyah	1.03 (0.44–2.40)		0.42 (0.10–1.71)		0.34 (0.09–1.27)	
Al-Ahmadi	1.92 (0.82–4.54)		0.42 (0.10–1.67)		0.98 (0.29–3.40)	
Al-Jahra	2.25 (0.98–5.61)		0.61 (0.15–2.53)		1.23 (0.35–4.34)	
Mubarak Al-Kabeer	2.32 (0.97–7.21)		0.41 (0.07–2.41)		2.40 (0.52–9.11)	
**Monthly income (KD)**		0.008		0.308		0.178
< 500	Reference		Reference		Reference	
500–1000	1.96 (1.07–3.59)		1.37 (0.65–2.90)		0.87 (0.31–2.41)	
> 1000	2.76 (1.42–5.36)		1.86 (0.84–4.08)		2.42 (0.74–7.91)	
**MetS**		<0.001		<0.001		<0.001
Yes	5.04 (3.13–8.13)		3.46 (1.87–6.37)		7.23 (3.41–15.32)	
No	Reference		Reference		Reference	

T2DM: Type 2 diabetes mellitus; CHD: Coronary heart disease; n: Number of participants; OR: Odds ratio; CI: Confidence interval; Yrs: Years; KD: Kuwaiti Dinars;

MetS: Metabolic syndrome.

## Discussion

To the best of our knowledge, this is the first study to estimate the 10-year risk for developing T2DM and CHD using the FINDRISK and FRS screening tools, and to determine the prevalence of MetS using the 2009 Joint Statement criteria among the general population in Kuwait. Results from the current study will identify adults at different levels of risk to develop T2DM and/or CHD risk in the next 10 years; hence it is valuable for policy makers and clinicians to inform future services. Furthermore, our results would be the first step in providing a quantitative measurement of the distribution of FINDRISK and FRS levels among the general population in Kuwait, and may hold promise for surveillance purposes.

The prevalence of smoking among respondents was low compared to previous studies from Kuwait [22, 45, 46]. The low prevalence in our study may be attributed to the exclusion of subjects aged < 20 years, and probably the high awareness among respondents of the health warnings in relation to smoking. In this study, smoking prevalence was significantly lower among females, which was most likely due to women in this conservative society not disclosing the fact that they smoke.

The prevalence of hypertension among the study population was comparable to that reported previously in Kuwait [37]. The current results identified that 8.4% of respondents had screen-detected hypertension and one third of the treated subjects had suboptimal control of BP. These participants remain at increased risk of micro- and macrovascular complications unless their BP can be adequately controlled at target BP goals. Thus, our findings highlight the need for public education to increase their awareness of the relevance of hypertension and the importance of regular BP monitoring and control. Furthermore, research is needed to understand the contributing factors that may lead to inadequate control of BP among patients with hypertension, such as failure to modify therapy when it is indicated as with combination therapy, patients’ failure to comply with recommended lifestyle interventions and poor medication adherence.

Over one-third of study subjects had high TC, which is an independent risk factor for CVD, and 2.6 million deaths worldwide were attributed to elevated TC [47]. In addition, about half of respondents had low HDL-C, which characterizes individuals with atherogenic lifestyle, because it is decreased by smoking, obesity and sedentary lifestyle [25]. Furthermore, the present study demonstrate that the combined prevalence of overweight and obesity is still high in Kuwait, which is a great public health concern due to their strong association with chronic diseases, including CHD, cerebrovascular disease, T2DM, dyslipidaemia and some types of cancer [48]. These findings can be explained by the dietary habits in Kuwait which are high in total calories, saturated fat and cholesterol, and low in vegetables, and by the sedentary lifestyle [49]. Cultural and lifestyle factors that promote sedentary lifestyle and lack of physical activity were reported as the main contributing factors to obesity in Kuwait [46]. The increase of wealth in Kuwait as an oil-producing country could possibly be another indirect contributing factor for obesity, T2DM and CVD [37]. In this regard, 52.7% of participants in the current study reported irregular consumption of fruit and vegetables, and about 40% did not have a daily physical activity of ≥ 30 minutes. These results underscore the need for urgent public health education, design and implementation of management strategies to control these modifiable risk factors.

The prevalence of IFG among respondents was found to be 24.4%, which is close to that in the United States (26.0%) [50] and Kuwait (21.5%) [39], but higher than that reported by the other three studies conducted in Kuwait between 2001 and 2009 (ranged between 8.0% to 15.1%) [35, 36, 51]. The latter can be explained by the small sample size or the use of the old ADA diagnostic criteria. The mean FPG for respondents in the current study was 5.3, which was lower than that reported by Al-Rashdan et al (5.6 mmo/l) [37] and Al-Zenki et al (5.9 mmol/l) [38], which is most likely due to the inclusion of patients with diabetes in their sample. The participants’ mean FPG was within the desirable level, although it was on the borderline-high level, and this underscores the need for effective lifestyle interventions. The prevalence of 4.0% for screen-detected T2DM among respondents was similar to that of previous studies in Kuwait (4.1%) [39], but lower than that reported in Hungary (28.7%), Mexico (20.5%) and India (10.5%) [17, 19, 52]. Individuals with undiagnosed T2DM and chronic hyperglycaemia are at significantly higher risk for stroke, CHD, and peripheral vascular disease than the healthy subjects [5, 6, 43] Thus, continuous screening and monitoring of plasma glucose is needed in Kuwait for early detection and prompt treatment, which will reduce the burden of diabetes and its complications.

Our findings identified that about one-third of respondents had MetS as defined by the 2009 Joint Statement criteria, which was lower than that from previous studies in Kuwait, conducted by Al-Rashdan et al in 2006 (36.2%) [37] and Al-Zenki et al in 2008–2009 (40.8%) [38] using the IDF criteria. It is expected that the prevalence will be higher using the 2009 Joint Statement criteria than that reported by the previous two studies. However; the decrease in prevalence of MetS in this study may be an indication for improvements in the lifestyle habits among participants, especially increased physical activity, since approximately 61.0% of them reported a daily physical activity ≥ 30 min. The present findings that the prevalence of MetS significantly increased with age, overweight, obesity and decreased physical activity are consistent with previous studies [37, 38, 53–55]. However; there was no significant difference of the MetS prevalence by gender, which is similar to that reported by Al-Rashdan et al [37]. The fact that about one-third of participants had MetS shows the importance of its assessment in primary care practice by considering a number of individual risk factors. This will help early identification of patients who are candidates for lifestyle interventions and control of the modifiable risk factors, which in turn will reduce the incidence of CVD and T2DM [32].

The present results estimated that about 30% of respondents were at either moderate, high or very high risk of developing T2DM within the next 10 years. This is similar to that reported in Saudi Arabia (29.4%) and Libya (32.2%) [13, 15], but lower than that in Hungary (41.0%), Cuba (74.4%) and among Pakistani immigrants in Norway (52.9%) [16, 18, 19]. Our finding of 13.2% of participants with high/very high 10-year risk of developing T2DM is comparable to studies from Saudi Arabia (10.7%) and Libya (12.3%) [13, 15], but lower than that reported in Norway (28.5%), Cuba (26.8%) and Mexico (28.9%) [16–18]. In this study, the risk for developing T2DM was higher among females compared to males, which is in agreement with studies in Libya, Hungary and Norway [13, 18], and least common among the youngest aged responders (20–44 years) that is consistent with previous studies [39, 51, 56]. Also the level of education, monthly income and MetS were found to be significantly associated with moderate/high/very high risk of developing T2DM, which is in accordance with previous studies [12, 51]. The present results highlight the need for more focused and intense health promotion campaigns, with the aim of reducing overall risk for T2DM. This will require a holistic public health approach of multifaceted programs targeting several risk factors. The prevention role of the National Diabetes Program in Kuwait should also be enforced, because prevention is the most efficient way to avoid the dramatic consequences of T2DM [10].

Approximately one-fifth of respondents were at moderate or high risk for CHD within the coming 10 years. The risk increased strongly with age of ≥ 55 years, and was higher among males than females. A similar trend has also been reported in the United States [26, 27], but different from Finland in which 9.0% of study population had a high risk of developing CHD [28]. Almost one-in ten responders had a moderate or high risk of developing both T2DM and CHD within the following 10 years. The current results underscore the importance of identifying the 10-year risk for CHD among people with ≥ 2 of the following risk factors (cigarette smoking, hypertension, low HDL-C, family history of premature CHD, and age) in the daily clinical practice [25]. Individuals with low, moderate, or high risk of developing CHD usually have one or more risk factors. It has been recommended that subjects with a low or moderate level of CHD risk should be informed about their increased risk and offered lifestyle modification counselling, while those with a high level of risk should have all risk factors addressed to reduce their risk of CHD and overall CVD [28].

Findings from this study should be useful to public health authorities, researchers and clinicians who have a role in the development of preventive and treatment guidelines for T2DM and CHD in Kuwait. The current results reflect some of the possible issues with current health promotion and disease prevention strategies. Since MetS, CHD and T2DM share common modifiable risk factors that are prevalent among the population in Kuwait, a recommendation is made for developing and implementing an organized delivery system to place priority on preventive services rather than focusing solely on treatment to reduce public health burden from these conditions. This could be achieved by appropriate and continuous risk screening for MetS, CHD and T2DM at primary care practices, active counselling, timely treatment by healthcare providers, the use of multifaceted interventions, and motivational health promotion campaigns. The cost-effectiveness of these interventions should be explored and policy makers should consider giving a higher priority for interventions that were supported by evidence to be cost-effective. Implementation of these may be potentially challenging in the primary health care settings due to the fact that patient care in Kuwait is mainly provided by physicians, who face time limitations in being able to implement these with everyone attending their clinic. There is evidence that supports health promotion interventions by non-physicians in the primary care setting [57–59]. Hence, a potential intervention could be the integration of an effective collaborative multi-disciplinary team approach, including physicians, pharmacists, nurses and dieticians to encourage patient education and self-care, and share responsibility for individuals achieving therapeutic goals [60]. Furthermore, the establishment and strengthening of an ongoing surveillance of 10-year risk estimation of developing T2DM and/or CHD and associated risk factors using FINDRISK and FRS in Kuwait will help in following-up the changes in prevalence of the risk factors, MetS, and estimates of the public in each risk stratum.

## Limitations

Interpretation of the findings of this study should take into account certain potential limitations that might impact upon the conclusions drawn. The major setback has been the use of a convenience sample. That would have affected the external validity in terms of generalizing the findings to the wider population. The data analyses showed the study sample had over representation of female gender and higher educational levels compared to the latest census data. With an aim of correcting for this selection bias, further sample adjusted weighted analyses were carried out. It was re-assuring to note that sample adjusted estimates were not much different from that of the unweighted analysis. Given this study was one of the largest conducted in Kuwait and included subjects from all governorates and suburbs the findings from this study is expected to ignite discussion on the risk of developing diabetes and CVD. Furthermore, some of the results obtained in this study were based on self-reported data about health behaviors; diagnosis of chronic diseases and medication use, which depends very much upon information given by respondents and is open to recall bias or error. Examining the extent of truthful answers or verifying respondents’ claims is not possible in this type of study, which were taken at face value. Finally, the cross-sectional nature of the data time that does not reflect any changes over time in relation to prevalence of risk factors and 10-year risk for T2DM and/or CHD. Findings from the current study should be confirmed with future longitudinal studies.

## Conclusions

The findings from this study provide important information about the prevalence of modifiable risk factors for T2DM, CHD and MetS, and estimates of the distribution of 10-year risk T2DM and/or CHD among the general population of Kuwait. These results allow for important comparative work with existing and future investigations in Middle Eastern countries. Our 10-year risk-stratification results showed that the use of such simple risk-prediction tools to promptly identify those individuals at risk is a valuable clinical strategy that should be more widely implemented in everyday primary care practice.

## Supporting Information

S1 Raw Dataset 10-Year Risk Estimation for T2DM and CHD in Kuwait. (SAV)
